# Physicians communicating with women at genetic risk of breast and ovarian cancer: Are we in the middle of the ford between contradictory messages and unshared decision making?

**DOI:** 10.1371/journal.pone.0240054

**Published:** 2020-10-08

**Authors:** Marta Fadda, Pierre O. Chappuis, Maria C. Katapodi, Olivia Pagani, Christian Monnerat, Véronique Membrez, Sheila Unger, Maria Caiata Zufferey

**Affiliations:** 1 Faculty of Biomedical Sciences, Institute of Public Health, Università della Svizzera italiana, Italiana, Switzerland; 2 Division of Genetic Medicine and Division of Oncology, Geneva University Hospitals, Geneva, Switzerland; 3 Faculty of Medicine, Department of Clinical Research, University of Basel, Basel, Switzerland; 4 Geneva University Hospitals, Geneva, Switzerland; 5 Service of Oncology, Hôpital du Jura, Delémont, Switzerland; 6 Division of Medical Genetics, Hôpital du Valais, Institut Central (ICH), Sion, Switzerland; 7 Service of Medical Genetics, Centre Hospitalier Universitaire Vaudois, Lausanne, Switzerland; 8 University of Applied Sciences and Arts of Southern Switzerland, Manno, Switzerland; CNR, ITALY

## Abstract

*BRCA1/2* genetic testing offers tremendous opportunities for prevention, diagnosis and treatment of breast and ovarian cancer. Women acquire valuable information that can help them to make informed decisions about their health. However, knowing one’s susceptibility to developing cancer may be burdensome for several women, as this risk needs to be managed over time through a continuous dialogue with multiple healthcare professionals. We explored how communication between physicians and unaffected women carrying *BRCA1/2* germline pathogenic variants was experienced by women in relation to their genetic risk. Data came from qualitative interviews conducted in Switzerland with 32 unaffected women carrying *BRCA1/2* pathogenic variants and aware of their genetic status for at least 3 years. We identified three different types of message as conveyed by physicians to women: (1) a normative message, (2) an over-empowering message, and (3) a minimizing message. On one hand, we found that women are exposed to contradictory messages, often simultaneously, in their interactions with healthcare professionals during their post-genetic testing journey. On the other hand, women’s reports highlighted the absence of shared decision-making in such interactions. The combination of these two findings resulted in a strong sense of disorientation, frustration, and powerlessness among participants. Healthcare professionals interacting with high cancer risk women are urged to align in favor of a both concerted and shared decision-making approach when discussing options for managing genetic risk.

## 1. Introduction

The identification of a gene predisposing to breast and ovarian cancer in 1994 (*BRCA1*) [1], and the subsequent discovery of a second gene related to the susceptibility to the same diseases (*BRCA2*) [2] in 1995 quickly marked a new era for genetic testing, providing women and their biological relatives with new opportunities for cancer risk assessment and management [3,4]. As increasingly affordable genomic sequencing technologies evolve and the benefits of new strategies become more evident for inherited cancers, we witness an increased demand and urgency for genetic testing [3].

Today, identification of susceptibility to breast and ovarian cancer through genetic testing offers tremendous opportunities not only for an early diagnosis of the disease but also its prevention and treatment [5,6]. However, despite improved medical and biological knowledge, genetic testing *“also raises numerous ethical and practical issues, both scientific and social, that must be addressed by the medical community”[1]*. Preventive healthcare focuses on healthy people and aims to identify risk factors potentially associated with the development of certain diseases. This approach has generated a new category of people: at-risk individuals, referred to as “partial patients” [7] or “patients-in-waiting [8], affected by a “proto-disease” [9] and living in a suspended space between health and illness [10]. In their 1994 Science paper, Miki et al. were already pointing at this ambivalent role and at the implications of patients’ construction and management of such at-risk status for their physical and psychological wellbeing, to which healthcare providers, family members, and society at large may contribute to.

Because of their high risk of developing cancer, unaffected women carrying *BRCA1/2* germline pathogenic variants perfectly represent this category of patients and illustrate the practical and ethical issues that their risk status entails. A prospective cohort study of 6,036 *BRCA1* and 3,820 *BRCA2* female mutation carriers estimated that 72% of women with a pathogenic *BRCA1* variant and 69% of women with a pathogenic *BRCA2* variant will develop breast cancer by the age of 80, while the cumulative ovarian cancer risk was found to be 44% for *BRCA1* carriers and 17% for *BRCA2* carriers [11]. It has been stressed that being aware of one’s susceptibility to developing breast/ovarian cancer may result in enhanced empowerment [12], as more options over prevention and surveillance are offered and, therefore, more control over one’s life. Women found to carry pathogenic variants have the possibility of adopting measures to minimize their risk, such as intensive surveillance to detect cancer as early as possible, or prophylactic surgery to dramatically reduce the risk of developing the disease [13]. In Switzerland, these strategies are offered to all at-risk women since basic universal health insurance covers surveillance and prevention measures for *BRCA1/2* carriers. At the same time, genetic testing and associated medical care are supposed to be optional. The Federal Act on Human Genetic Testing ensures the individual’s *right not to know* their genetic risk or opt for no further medical intervention in the event that a mutation is found (Art. 6 and Art. 18, Federal Act on Human Genetic Testing, 2014) [14].

Despite these positive aspects, discovering one’s susceptibility to developing breast and ovarian cancer may imply important challenges for women. In particular, deciding how to manage genetic risk may be a demanding task because of its probabilistic nature and the implications of the different surveillance and preventive measures (e.g. potential harms of mammography, possible complications of preventive surgery, premature menopause, etc.). Moreover, evidence suggests that individuals do not always optimally understand the meaning of cancer screening and, therefore, may have difficulties in making decisions [15,16]. In a previous study [17] we showed that Swiss unaffected women carrying *BRCA1/2* pathogenic variants experience disorientation regarding their health status, their specific duties and rights within the healthcare system, how they are supposed to behave as “at-risk” individuals and how they should make decisions.

In light of such challenges, it is hard to overstate the importance of communication with the healthcare professionals in fostering women’s informed medical decision-making. However, communication may turn out to be a double-edged sword and raise problems. Managing genetic risk requires complex teamwork, as unaffected women are supposed to interact with several health professionals during their post-genetic testing journey, but coherence and coordination between them are not granted. This is even more likely in Switzerland, where unaffected, at-risk women are often managed by physicians in private practice, potentially lacking the coordination that may characterize a hospital setting. Until recently, genetic testing was performed in specialized counseling centers within hospitals. Once a pathogenic variant was identified, women were discharged by the hospital where they had been initially screened and usually returned to their primary-care physicians. Today, the role of private practice has become even more relevant, as novel technologies have made genetic testing easier, faster and more affordable. Consequently, genetic tests are increasingly ordered and managed by primary care providers [18]. Studies conducted in similar contexts found that primary care providers (either with or without previous genetic training) do not consider themselves knowledgeable about the genetic basis for common diseases nor feel prepared for working with patients who have had genetic testing for common diseases or are at high risk for genetic conditions [19–21].

Starting from the assumption that communication during the post-genetic testing journey is crucial and potentially problematic, the goal of this study was to explore the kind of messages healthcare providers convey, consciously or unconsciously, to unaffected women carrying germline *BRCA1/2* pathogenic variants, from the perspective of the women themselves. This paper results from analyses of interview data collected as part of a larger study conducted in Switzerland between 2011 and 2014 to explore the way unaffected mutation carriers manage their cancer risk over time.

## 2. Materials and methods

### 2.1. Study design

The study adopted a grounded theory approach, a specific qualitative research method used in social sciences that involves the collection of narratives from participants to understand and conceptualize their experience and to investigate how this is influenced by their social context. Grounded theory is particularly suitable to investigate complex and underexplored phenomena and aims to generate theories that are grounded in the data, rather than test an existing theory. Following the Grounded Theory design, we conducted face-to-face interviews to understand women’s management of their breast and ovarian cancer risk [17]. In recent years, then, the authors’ scientific interests have shifted towards the subject of communication, which led to resuming the collected material and deepen the analysis through this particular lens.

### 2.2. Recruitment

After approval by local ethics committees, the authors contacted potential participants through four genetic-counseling hospital services based in the French and Italian parts of Switzerland between 2011 and 2014. To be eligible for the study, participants had to be unaffected female carrying *BRCA1/2* pathogenic variants discovered at least three years before the interview; according to the study team, a timespan of three years was adequate to gain some experience with living with genetic risk. During the recruitment period, the genetic-counseling centers identified 53 women who met all inclusion criteria. All of them were contacted by the centers and 32 women (response rate 58%) accepted to take part in the study (Table 1).

**Table 1:** Participants’ recruitment.

53 women contacted by the genetic counseling services (HUG: 21, CHUV: 20, RSV: 7, EOC: 5)					1 woman contacted the researcher directly after having heard about the study (HNE)
31 accepted	14 never answered	5 refused to participate (lack of time, no change in lifestyle, no experience to share, considered her experience private, lived abroad)	2 could not be reached	1 accepted but could not be reached afterwards	
32 women included in the final sample (HUG: 8, CHUV: 15, RSV: 4, EOC: 4, HNE: 1)					

Abbreviations: CHUV, Centre Hospitalier Universitaire Vaudois; HNE, Hôpital Neuchâtelois; HUG, Hôpitaux Universitaires de Genève; EOC, Ente Ospedaliero Cantonale; RSV, Réseau Santé Valais.

### 2.3. Sample and data collection

The sample was composed of 32 participants whose age ranged between 26 and 60 years (M = 41), whereas the time elapsed since genetic testing ranged between 3 and 12 years (M = 6). Table 2 shows participants’ socio-demographic and clinical characteristics. Participants adopted a broad range of measures to deal with their at-risk status, which were described in a previous article [17]. Data were collected through one or two retrospective, biographical interviews and through documents that participants had accumulated over their lifespan and shared with the research team, such as copies of medical letters and notes. The interviews lasted approximately 2 to 3 hours in total, they were audio recorded and transcribed verbatim. Questions covered multiple aspects related to the management of participants’ genetic risk over time and to the relationships with healthcare providers and in general with the healthcare system.

**Table 2:** Study participants.

Socio-demographic and clinical characteristics	n = 32
Age (years)	
26–35	n = 8
36–49	n = 21
50–60	n = 3
Education	
Secondary education	n = 19
University education	n = 13
Children	
No children	n = 7
Had children before the testing	n = 14
Had children after the testing	n = 11
Language	
French	n = 28
Italian	n = 4
Living area	
Urban	n = 18
Rural	n = 14
Marital status	
Married	n = 26
Single	n = 3
Divorced	n = 2
In a relationship	n = 1
Years elapsed since genetic testing	
3–6	n = 9
7–12	n = 23
Undertaken measures	
Breast surveillance	n = 1
Breast + ovarian surveillance	n = 9
Prophylactic bilateral annexectomy + breast surveillance	n = 12
Prophylactic bilateral mastectomy + ovarian surveillance	n = 4
Prophylactic bilateral annexectomy + mastectomy	n = 6

### 2.4. Analysis

To analyze the data we adopted an inductive approach guided by constant comparison, which was facilitated by the software Atlas.ti [22]. One of the authors (MCZ) categorized the data into broad categories and then examined the similarities and differences in participants’ accounts in order to summarize their experiences by engaging, at the same time, in regular discussions on emerging thematic patterns with the other authors.

## 3. Results

Previous analyses of the present dataset revealed that participants expressed a sense of disorientation when describing their experience of living with and trying to cope with genetic risk [17]. In particular, decision-making to manage genetic risk was reported as extremely painful. The present study, specifically focused on communication, showed that the exchanges between women and the healthcare providers they encountered after their genetic testing played a pivotal role in this context. During their risk management trajectory, which started with the disclosure of women’s genetic testing results and continued throughout the rest of their lives, participants met a significant number of practitioners. Analysis of the interviews especially revealed contacts with gynecologists and radiologists, but also with surgeons, endocrinologists, medical oncologists, GPs, dermatologists and ophthalmologists, all of them being involved in some way in the management of women’s genetic risk. On average, every participant reported having been in regular and simultaneous contact with four different physicians over their post-genetic trajectory. No gender or institutional (public hospital or private practice) prevalence emerged. According to most participants, messages delivered by these different health professionals lacked consistency. These messages were conveyed either explicitly–through words–or implicitly–through attitudes or behaviors. Through the analysis of participants’ reports, we identified three different types of message summarized as follows: (1) a normative message, (2) an over-empowering message, and (3) a minimizing message. Participants reported to have received more than one type of message by healthcare providers encountered after their genetic testing. Being a qualitative study with a sample of 32 women, we were not able to identify a predominant message with any statistical significance. However, it may be worth pointing out that the normative message was more frequently reported, followed by the minimizing one.

### The normative message

Many participants reported they were often asked to adopt risk-management behaviors consistent with international guidelines, such as prophylactic risk-reducing surgery. Women reported that, occasionally, healthcare providers were more flexible and simply encouraged them through invitations or suggestions. Sometimes health professionals openly pressured to make them adhere to their medical recommendations. Nikita, 38 years old, performed her genetic testing at 31 and had her breasts removed at 37. She was very resistant to the idea of removing her ovaries, but her gynecologist told her explicitly that she could not escape surgery:

*“Every time*, *he [the gynecologist] tells me that he’s not going to let me cross 40 years with my ovaries*. *He says*: *<<Take your time*, *but you will have to remove them>>”*. (Nikita, 38)

Another participant, Attilia, was 42 years old at the time of the interview and tested when she was 37. She did not want to remove her breasts or ovaries, but her gynecologist did not agree with her:

*“My gynecologist would like me to decide immediately for both the interventions*. *<<It's foolish to wait for the disease>>*. *She said that to my face*. *She said*: *<<I don't know what's better*, *whether to have the operation or to have the disease*. *Think about it*. *Because with the disease*, *you know when you're going in*, *but you don't know when you're going out>>*.*”* (Attilia, 42)

These quotations showed that some participants had the feeling they were asked or were obliged to adhere to international guidelines, and if they resisted, they felt irresponsible or irrational. Alix expressed this feeling of obligation and her resulting frustration very clearly. Because she was willing to undergo medical exams only once a year, she regularly had to argue with her radiologist:

*“I don’t want to think about cancer all my lifetime*. *I want to feel good in my skin for 11 months and 25 days in a year*. *Before the exam*, *I suddenly dream that I have a breast growing up on my back*, *or that I break the imaging machine*, *this kind of things*. *Or I think <<that’s it*, *this time they are going to find something>>*. *Thus*, *I agree to have 5 days in a year that I continuously think of cancer*. *That’s it*. *And he [the radiologist] cannot understand*. *[…] I have to fight not to do an exam despite I'm in good mental health*. *Well*, *I can demand not to do it*, *don’t I*?!?” (Alix, 45)

### The over-empowering message

Some participants reported that the healthcare providers invited them to ask themselves what they considered important for their health, and to make decisions accordingly. These physicians maintained a completely neutral attitude, resulting in participants feeling they had the ultimate responsibility of their risk management behaviors and that it was up to them to make the final decision. For Elin, 45, who screened at 38 and had her breasts and ovaries removed at 40 and 43, respectively, it was difficult to decide for her prophylactic mastectomy. She reported spending months to figure out what to do, searching information and trying to understand her physician’s opinion on the best way to go forward. Regarding the latter, she reported becoming extremely frustrated when she realized that he did not want to give her his opinion:

*“He kept telling me it was up to me*, *that he could not put himself in my shoes*. *I told him*: *<<But what about if I were your sister*?*>> His answer was always*: *<<Look deep inside*, *talk about it*, *talk to your husband*, *it's up to you to decide*, *and I'll be there to do what you decide>>*.*”* (Elin, 45)

Another example of this type of attitude is presented by Désirée, 42, who had her genetic testing at 38 and her ovaries removed at the same time. Right after the testing result, Désirée was thinking of having preventive surgery. During a consultation with her gynecologist, she introduced the topic. His answer did not offer much room for discussion:

*“He said*: *<<Look*, *this is my position*: *it is your choice*, *it is your body>>*. *<<Okay>>*, *I told him*, *<<then I'm using you as a manpower*: *I would like you to do the intervention because you know me*, *I want you to do the surgery>>*. *And we planned the date*.*”* (Désirée, 42)

Later in the interview, Désirée stressed the concept of “manpower”: in her post-genetic testing journey, she used to consider her gynecologist as an instrument able of performing strictly technical tasks, but not as an interlocutor with whom to discuss, confront and make shared decisions.

### The minimizing message

Other participants explained they had the feeling that their genetic condition was not taken seriously by healthcare providers. Celesta, 32 years old, who did her genetic testing at 25 and had her ovaries removed at 33, reported to be very diligent in making appointments for surveillance after she discovered her cancer predisposition. However, her surveillance plans were not always supported by her healthcare providers. Because she had not developed cancer yet, but was “simply” susceptible to it, she was classified as “not urgent” and thus had difficulties in getting her check-ups done according to the planned schedule:

*“It’s like with your dentist*: *if you don’t have cavities*, *if you just want to go to the dental hygienist*, *then they’ll give you an appointment in three months*. *But if you say*: *<<I can’t stand it anymore*, *I have an abscess>>*, *they’ll find you an appointment*, *they’ll cancel the appointment for the person who isn’t sick in order to treat the person who is*.*”* (Celesta, 33)

In this case, it was stressed that the person was not sick. Other participants, like Gemma, 39, had a similar experience. Gemma underwent genetic testing at 36 and had her ovaries removed at 38. Since she did not have her breasts removed, she needed regular prescriptions for mammograms. However, she often found it difficult to convince her physician to fill out the prescriptions for her:

*“It is as if one had to beg for mercy*, *I have to beg for the prescription for mammogram*.*”* (Gemma, 39)

Avril, 42, who tested at 38 and had her ovaries removed at 39, reported to feel as a “second-class patient”. Every time she had a mammogram, she had the feeling that she was not considered a priority by the secretary of her radiologist:

*“The radiologist couldn’t give me the results immediately*, *so I had to call back*, *and the secretary sent me packing and said*: *<<Well*, *he doesn’t have time*. *He’ll call you back>>*. *I think that the people who work in this field don’t understand our burden*. *It was awful*. *I think this is a mistake*, *really; I think that there’s a mistake in the system*.*”* (Avril, 42)

## Discussion

Women carrying pathogenic *BRCA1* or *BRCA2* variants have different opportunities to manage their cancer risk. However, due to many implications such opportunities have for their health (for example, the burden of premature menopause following prophylactic oophorectomy), these women may have difficulties in making choices about whether to opt for enhanced screening or undergo preventive surgery, or about which surgery to have and when [23]. Dove and colleagues have raised concerns on how women interpret international guidelines and recommendations, and whether their interactions with healthcare professionals reflect the individualistic understanding of autonomy that *“people are*, *in their ideal form*, *independent*, *self-interested and rational gain-maximizing decision-makers”* or a more relational approach to patient autonomy [24].

The U.S. Preventive Services Task Force (USPSTF) and the American Cancer Society guidelines recommend that *“physicians individualize decisions about breast cancer screening and engage patients in shared decision making*” [25]. According to the shared decision-making approach, healthcare providers are expected to discuss together with at-risk women how to manage their cancer risk, so that clinical decisions are at the same time a reflection of patients’ preferences and values and in line with evidence-based practice [25]. Our study explores the communication process and pitfalls between at-risk women and their physicians. Our results are twofold: on the one hand, it appears that women are exposed to contradictory messages due to the lack of coherence and coordination among the various healthcare providers. A second finding is that women are far from being consistently engaged in shared decision-making.

**Fig 1:**
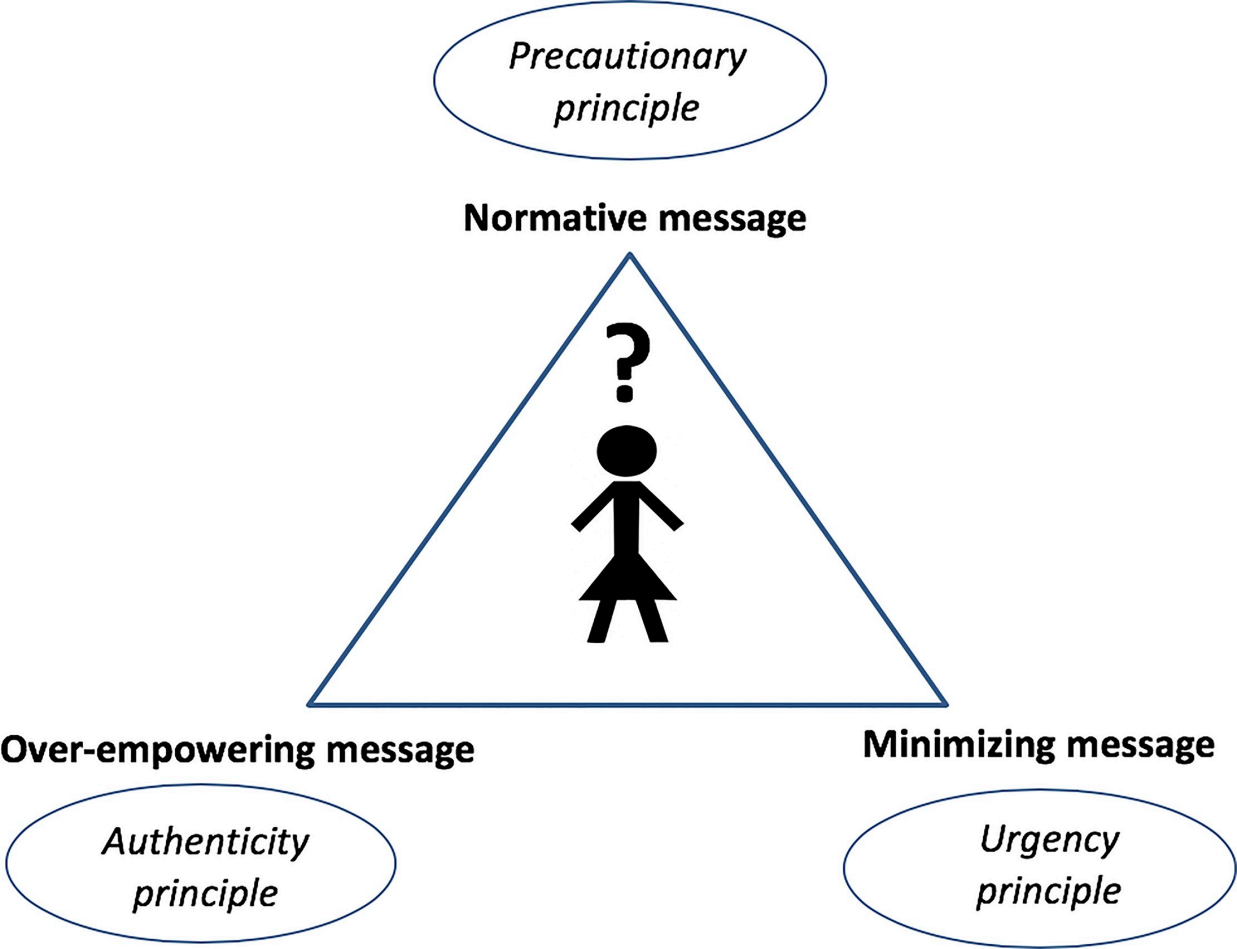
Disorientation triangle. Fig 1 illustrates the feeling of disorientation at-risk women experience after being exposed to the three different messages and their respective principles, which are represented as a triangle surrounding them. The three different types of message are: (1) a normative message (based on the precautionary principle), (2) an over-empowering message (based on the authenticity principle), and (3) a minimizing message (based on the urgency principle).

Regarding the first finding, the three types of messages delivered by the physicians that we extracted from participants’ reports are not novel as they are based on different assumptions well known in the medical realm. Concerning the normative message, the literature on preventive medicine has underlined the existence of the supremacy of disease avoidance over disease treatment (*“prevention is better than cure”*). The basic argument used or implied by physicians to persuade participants to adopt risk-management behaviors is the precautionary principle, suggesting that genetic information may be considered as a kind of weapon [26], and that it would be irrational not to use it to fight the disease risk [27]. The over-empowering type of message may be brought back to the authenticity principle, which typically guides doctor-patient interactions based on the informed model [28]. This model involves a partnership between doctor and patient based on a division of labor: the doctor communicates any relevant information on all available options, including their benefits and risks, and the patient makes an informed treatment choice, bearing the ultimate responsibility. Calling for a principle of authenticity, women were invited to *“look deep inside”*, as if the best decision could only be gut-driven rather than mutually agreed upon. An opportunity or urgency principle might be driving the minimizing message, showing that genetically at-risk women have no priority over other patients as they are, in fact, not sick yet. Fig 1 illustrates the feeling of disorientation at-risk women experience after being exposed to the three different messages and their respective principles, which are represented as a triangle surrounding them. The lack of a concerted approach/attitude among physicians who communicate with at-risk women is also in line with previous findings reporting that the point of view of the medical community is not unanimous towards preventive medicine [27,29]. The fact that genetically at-risk women may be exposed to contradictory messages by their healthcare providers makes the association between genetic knowledge and empowerment even more complex than expected, highlighting the key role physicians play in this regard during the post-genetic testing journey. While we might be tempted to see an immediate connection between awareness of one’s cancer risk and empowerment, our findings show that knowledge–if not managed through a consistent approach among all parties involved–may actually result in losing control (or power) over one’s life.

Regarding the second finding, the three types of messages all reflect interpretations of patient autonomy that are not consistent with the shared decision-making approach that has been recently called for in similar contexts (i.e. breast cancer screening) [25]. The three messages either suggest a paternalistic approach according to which it is the responsibility of the physicians to make the best decisions for their patients (normative and minimizing messages) or an individualistic approach according to which women should make their own decision alone (over-empowering message), disregarding the relational interpretation of autonomy that is the core of the shared decision-making model [24,30–32]. Shared-decision making can be time and resource consuming for physicians. Furthermore, patients are extremely heterogeneous in terms of communication preferences and tailoring the interaction according to each patient might be a difficult task. However, research has shown that creating a partnership with patients through the assessment of their values and preferences against the available evidence on disease prevention and treatment may be beneficial for patient-reported health outcomes [33].

Our results have to be considered in light of the study’s limitations. In particular, the data have been collected some years ago. As qualitative research is context-dependent [34], it is worth asking if these results are still relevant today. Undoubtedly, healthcare providers are more informed about basic genetics nowadays. Additionally, more and more of them in private practice are involved in ordering genetic testing and managing genetic risk today [19]. Being trained on genetics or more informed about genetic opportunities, however, does not necessarily mean that healthcare providers are able to integrate effectively this knowledge into their medical practice [20,21]. Additionally, the interpretation of genetic findings is even more complex now than in the past due to the high number of genetic variants discovered [35]. The popularization and democratization of genetic testing, then, have made communication an even more crucial issue. We believe, thus, that despite the changes that occurred in the past five years, these data still provide meaningful information to elucidate the nature of communication during the post-genetic testing journey of unaffected women carrying germline *BRCA1/2* pathogenic variants today.

On the basis of these considerations, some evident implications are worth noting. From a mere practical standpoint, a concerted approach should be promoted to improve communication and consistency not only between the healthcare providers and the patient, but also among the healthcare providers themselves. Considering that over the next five years there will be more genome sequencing performed in clinical than in research settings [36], it would be beneficial to design and implement a professional role operating to amalgamate the system and coordinate all the medical professionals involved in women’s post-genetic testing journey, accompanying the patient over time, and ensuring the adoption of a shared decision-making approach by all parties. *Ad hoc* training for primary care physicians or other healthcare professionals, e.g. genetic nurses, is necessary to improve their understanding of genetic principles and to promote an open and effective communication with patients according to the shared decision-making approach.

Some research implications have also to be mentioned. Recent studies have urged for more data on the factors and mechanisms that guide genetic counselling practice and on the outcome measurements to be used to establish best practices in meeting patients’ needs. In answering the call for more evidence, our study has set patient-provider communication as a priority research area in the strive to inform, guide and help to shape future genetic counselling practice. We, in turn, call for more research on how communication between genetically at-risk women, their family, and their healthcare providers influences women’s risk-management behaviors and how the adoption of a shared decision-making approach can be facilitated in order to be not only more efficient and feasible but also increasingly effective in improving patients’ clinical and psychological outcomes.
